# Alcohol Interacts with Genetic Alteration of the Hippo Tumor Suppressor Pathway to Modulate Tissue Growth in *Drosophila*

**DOI:** 10.1371/journal.pone.0078880

**Published:** 2013-10-21

**Authors:** Anoj Ilanges, Maryam Jahanshahi, Denis M. Balobin, Cathie M. Pfleger

**Affiliations:** 1 Department of Oncological Sciences, The Icahn School of Medicine at Mount Sinai, New York, New York, United States of America; 2 Yale University, New Haven, Connecticut, United States of America; 3 The Graduate School of Biomedical Sciences, The Icahn School of Medicine at Mount Sinai, New York, New York, United States of America; 4 Columbia University, New York, New York, United States of America; Instituto Gulbenkian de Ciência, Portugal

## Abstract

Alcohol-mediated cancers represent more than 3.5% of cancer-related deaths, yet how alcohol promotes cancer is a major open question. Using *Drosophila*, we identified novel interactions between dietary ethanol and loss of tumor suppressor components of the Hippo Pathway. The Hippo Pathway suppresses tumors in flies and mammals by inactivating transcriptional co-activator Yorkie, and the spectrum of cancers associated with impaired Hippo signaling overlaps strikingly with those associated with alcohol. Therefore, our findings may implicate loss of Hippo Pathway tumor suppression in alcohol-mediated cancers. Ethanol enhanced overgrowth from loss of the *expanded*, *hippo*, or *warts* tumor suppressors but, surprisingly, not from over-expressing the *yorkie* oncogene. We propose that in parallel to Yorkie-dependent overgrowth, impairing Hippo signaling in the presence of alcohol may promote overgrowth via additional alcohol-relevant targets. We also identified interactions between alcohol and Hippo Pathway over-activation. We propose that exceeding certain thresholds of alcohol exposure activates Hippo signaling to maintain proper growth control and prevent alcohol-mediated mis-patterning and tissue overgrowth.

## Introduction

Alcohol consumption is a significant risk factor in cancers of tissues that contact or metabolize alcohol (for example, upper aerodigestive tract and liver cancers) and also in other tissues such as breast cancer [1-16]. Alcohol-associated cancers are responsible for more than 3.5% of all cancer deaths, yet how alcohol causes cancer remains a major open question. *Drosophila* are amenable to rigorous functional genetic analysis and descriptive phenotypic characterizations. *Drosophila* models have been established to explore the role of alcohol in health contexts including models of fetal alcohol syndrome and alcohol addiction [17-23] as well as cancer-relevant phenotypes such as tissue overgrowth [24-26] in a whole-animal model making it a particularly relevant system to investigate the relationship between alcohol and cancer where systemic responses could underlie the pathogenesis of disease.

We identified novel interactions between dietary ethanol and the Hippo Tumor Suppressor Pathway, a signaling network highly conserved from flies to mammals. The Hippo Pathway acts as a master regulatory pathway to restrict growth and proliferation and to promote apoptosis, and its disruption is implicated in a number of cancers [27-34]. Hippo (Hpo; Mst1 and Mst2 in mammals) [35-39] is the upstream kinase in a core cassette in which activated Hpo kinase associates with Salvador (Sav1 or hWW45 in mammals) [40,41] and phosphorylates and activates downstream effector kinase Warts (Wts; Lats1 and Lats2 in mammals) [40,42,43] and Wts co-activator Mats (Mob1 in mammals) [44]. Wts phosphorylates and inhibits transcriptional co-activator Yorkie (Yki; YAP and TAZ in mammals) [45], a potent oncogene. Components of this core cassette can be regulated by distinct upstream factors to define the eventual biological outputs. For example, GPCR signaling regulates Wts directly [46], Sik kinases regulate Sav [47], and a set of FERM-domain proteins including Merlin (Mer) and Expanded (Ex) act upstream to activate Hpo by an as yet undefined mechanism [48]. 

We found that alcohol exposure enhanced overgrowth upon Hippo Pathway attenuation in multiple organs in *Drosophila*. Surprisingly, alcohol did not enhance overgrowth from over-expressing *yki*, suggesting the Hippo pathway may target *yki*-independent growth regulators that are alcohol-responsive. We also found that high doses of alcohol enhanced phenotypes of *hpo* over-expression. Our studies reveal multiple interactions between alcohol and the Hippo Pathway and suggest a previously undescribed role for Hippo signaling to prevent tissue overgrowth upon alcohol exposure.

## Materials and Methods

### *Drosophila* tools

RNAi was achieved using inverted repeat alleles from the Transgenic RNAi Project for *hpo* (P{TRiP.JF02740}attP2, referred to here as *hpo* ^*IRT*^), for *Mer* (P{TRiP.JF02841}attP2, *Mer* ^*IRT*^), and for *ex* (P{TRiP.JF03120}attP2, *ex* ^*IRT*^), and from the National Institute of Genetics for *wts* (12072R-2, *wts* ^*IRN*^) and for *yki* (4005R-2, *yki* ^*IRN*^). Mosaic analysis utilized the FLP/FRT system [49] including the stocks *y w eyFLP; FRT42D pW+ UbiGFP/SM6-TM6B* and w; *FRT42D hpo* ^MGH1^. Over-expression of transgenes used stocks *UAS ykiV5*, *UAS FLAGyki*, *UAS ykiS168AGFP.HA*, and *UAS hpo*. Screen crosses and other experimental crosses were reared on Formula 4-24 Blue food (Carolina Biological) reconstituted from 1 gram of food flakes per 4 milliliters of the specified doses of ethanol or water. Previous studies established that alcohol doses in food decline over time [50]; we exploited this to try to recapitulate the human scenario of periodic alcohol exposure, not constant alcohol exposure, food in each vial was supplemented with 300 microliters of ethanol at the specified dose every other day. 

### *Drosophila* alcohol screen

To identify interactions between alcohol and overgrowth models, or alcohol and alterations in signaling pathways, we utilized both (1) Gal4/UAS [51] and (2) FLP/FRT systems [49] to generate contexts of tissue overgrowth in an ongoing screen (1). We crossed tissue-specific gal4 drivers (including wing drivers *engal4*, *c765gal4*, eye driver *eygal4*, etc.) to UAS transgenes that direct RNAi or over-expression of genes known to cause tissue overgrowth or participate in signaling cascades on food sources containing a range of ethanol or water only. We considered positive hits those genes whose overgrowth phenotypes were enhanced when larvae were reared in the presence of ethanol (2). We crossed *eyFLP*-containing stocks to corresponding FRT chromosomes containing mutations that cause tissue overgrowth. We considered positive hits those mutations whose over-representation in a mosaic eye was enhanced when larvae were reared in the presence of ethanol.

### Genotypes

**Figure 1:**
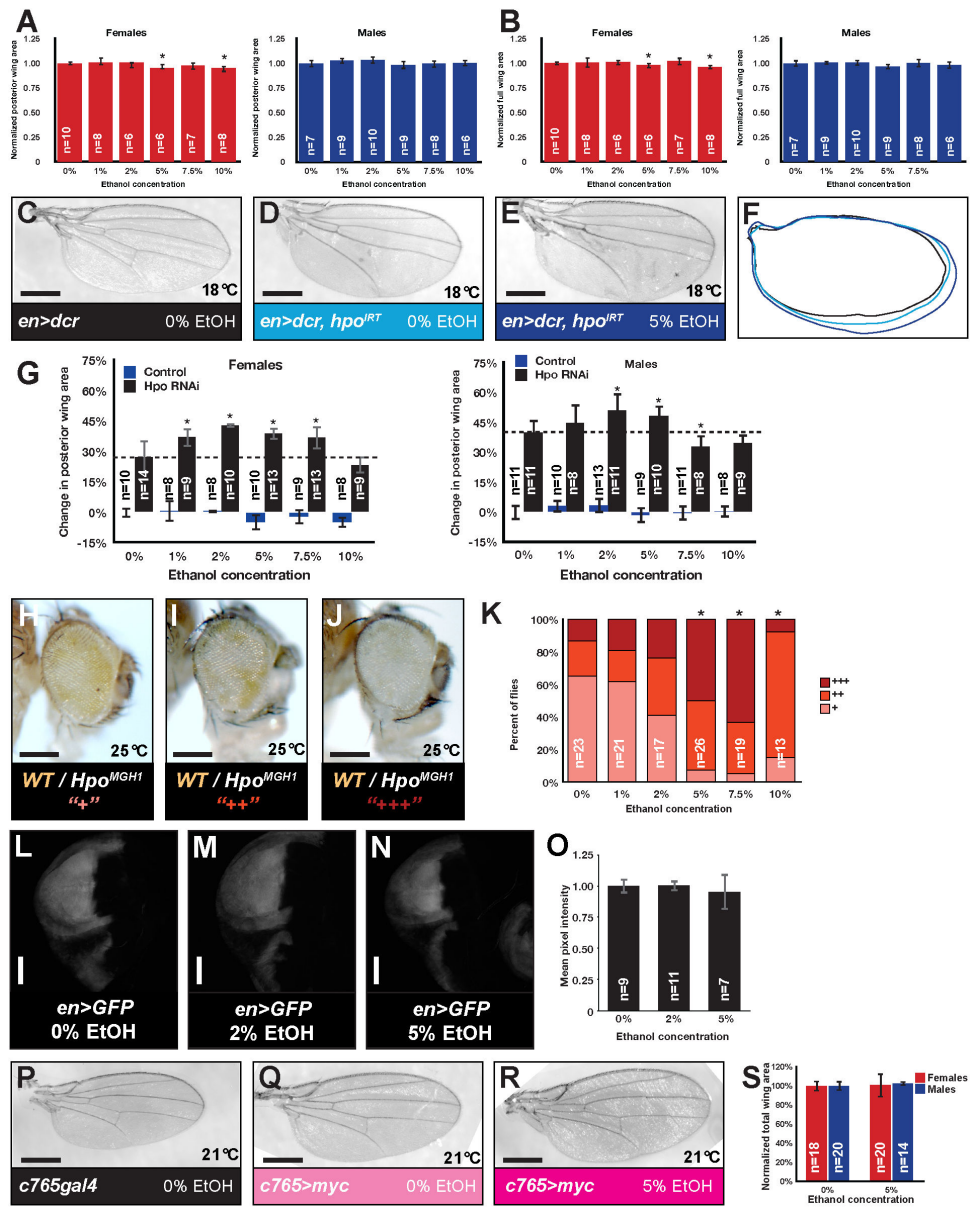
Dietary ethanol enhances overgrowth associated with loss of *hpo*. (A-B) Quantification of the effects of 1-10% ethanol on the posterior wing area (A) and whole wing area (B) of control flies (normalized to area of control wings on 0% ethanol). * indicates p=0.023995 for females at 5% and p=0.000633 for females at 10% in A and p=0.016029 for females at 5% and p=0.001122 for females at 10% in B. (C) Control wing. (D) *hpo* RNAi in the posterior wing. (E) Ethanol-mediated enhancement of wing overgrowth upon *hpo* knockdown. (F) Wing tracings from C-E highlight size changes. C-E, representative female wings. (G) Graph showing percent change in posterior wing area compared to controls at 0% ethanol for RNAi to *hpo* (black) and controls (blue) for food containing 0%, 1%, 2%, 5%, 7.5%, and 10% ethanol (normalized to areas of control flies treated with 0% ethanol). * indicates p=0.00224 (1%, females), p=0.00979 (2%, females), p=0.00589 (5%, females), p=0.00104 (7.5%, females), p=0.00730 (2%, males), p=0.02938 (5%, males), and p=0.01457 (7.5%, males). (H-J) Mutation in *hpo* (*hpo^MGH1^*) results in over-representation of mutant tissue (white) compared to wild-type tissue (red) [50]. Mosaic eyes fall into a range of over-representation from the mildest ratio scored as “+” (H) to moderate “++” (I) and severe “+++” (J). (K). Ethanol enhanced over-representation of mutant tissue in a mosaic eye, noted by the increase in “++” and “+++” eyes. This phenomenon was observed most strongly at 5% and 7.5% ethanol. Wild-type tissue in a mosaic eye serves as an internal control. We saw no gender differences; graph represents combined data for males and females. * indicates p=1.33E-10 (5%), p=3.97E-11 (7.5 %), and p=8.1E-6 (10%) compared to the same genotypes on 0%. (L-N) Images of wing discs expressing a GFP transgene under the control of *engal4* in flies reared on food containing (L) 0% ethanol, (M) 2% ethanol, and (N) 5% ethanol. Scale bar on wing disc images reflects 100 micrometers. (O) Graph reflecting quantification of GFP based on pixel intensity shows no change in GFP in flies reared on ethanol (p>0.05). (P) Control wing. (Q) *Myc* over-expression across the wing from a fly grown on control food. (R) *Myc* over-expression across the wing from a fly grown on food containing 5% ethanol. (S) Graph showing wing area for flies reared on 5% ethanol compared to controls at 0% ethanol for myc over-expression. Wing area is normalized to 100% for myc-expressing flies reared on 0% ethanol. Genotypes for this and subsequent figures are detailed in Materials and Methods. The number of flies analyzed in each experiment in this figure and subsequent figures is shown in parentheses on the base of each column. The bars in each graph in this figure and subsequent figures indicate Standard Deviation.

**Figure 2:**
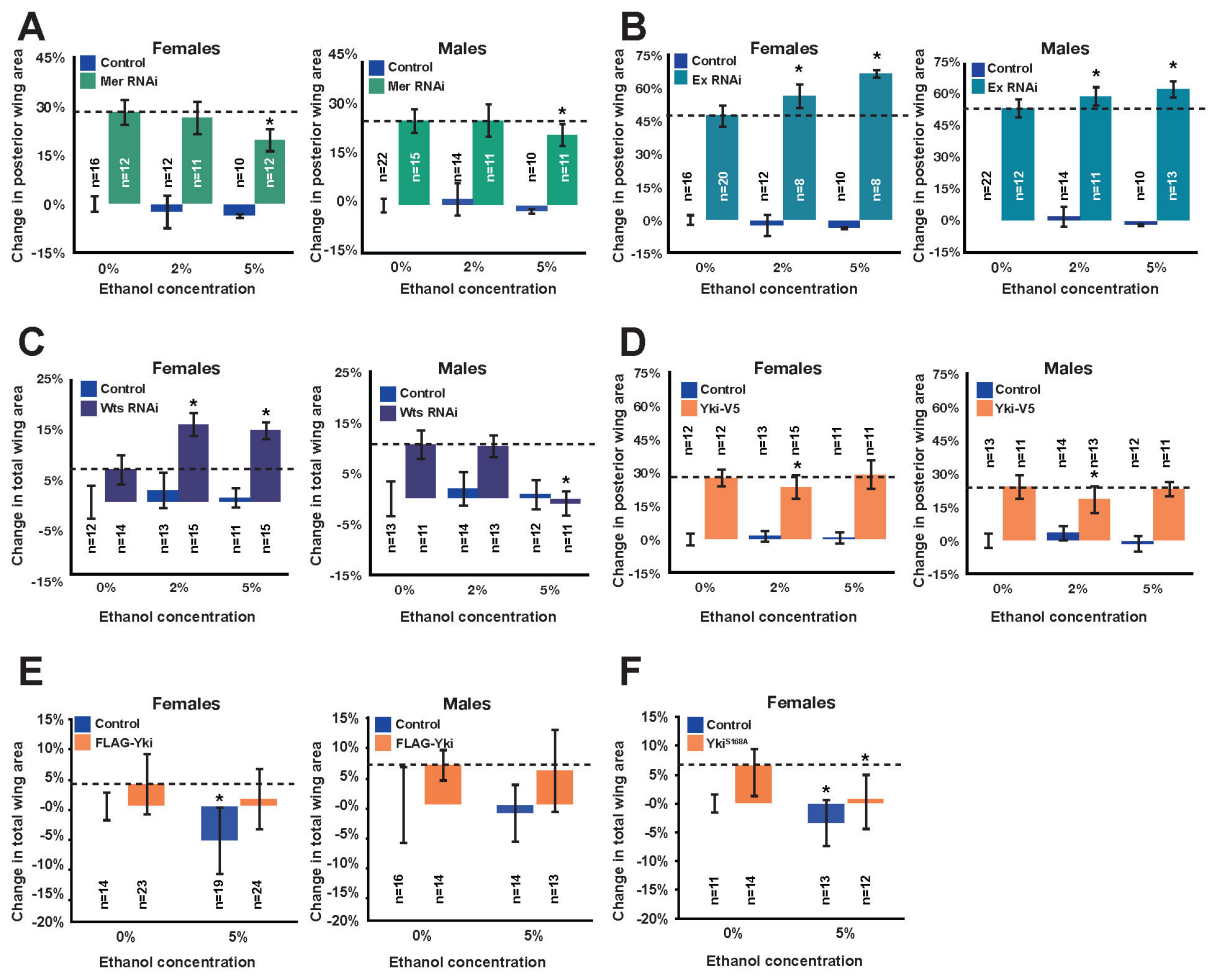
Ethanol enhances wing overgrowth associated with loss of Expanded, and Warts but not over-expression of Yorkie. (A-F) Percent change in posterior wing area (A-B, D) or total wing area (C, E-F) normalized to controls (*en>dcr2* for A-B, *c765gal4/+* for C, E-F, *engal4/+* for D) at 0% ethanol was graphed for each gene and for controls for food containing the indicated doses of ethanol (0%, 2%, and/or 5%) ethanol for (A) *Mer* RNAi in the posterior wing (* indicates p=1.08025E-05 for females at 5%, p=0.02382138 for males at 5%), (B) *ex* RNAi in the posterior wing (* indicates p=3.74229E-10 for females at 2%, p=0.005567613 for males at 2%, p=0.000441271 for females at 5%, p=3.00164E-05 for males at 5%), (C) Wts RNAi in the wing (* indicates p=1.64464E-7 for females at 2%, p=8.18059E-7 for females at 5%, p= 8.0633E-11 for males at 5%), (D) *ykiV5* over-expression in the posterior wing (* indicates p=0.004919 for females at 2%, p=0.041432 for males at 2%), (E) *FLAGyki* over-expression in the wing (*indicates p=0.000884273 for control females at 5%), and (F) *ykiS168A* over-expression in the wing (* indicates p= 0.00038782 for *ykiS168A* females at 5%; also, in this experiments, * indicates p=0.012339878 for female control wings at 5% compared to control wings at 0%). P values are given for comparisons of the same genotypes reared on the indicated doses of ethanol to those reared on control food lacking ethanol. Experiments in A-E were conducted at 21°C, experiments in C-D were conducted at 25°C, experiments in E were conducted at 18°C, and experiments in F were performed at 16°C.

**Figure 3:**
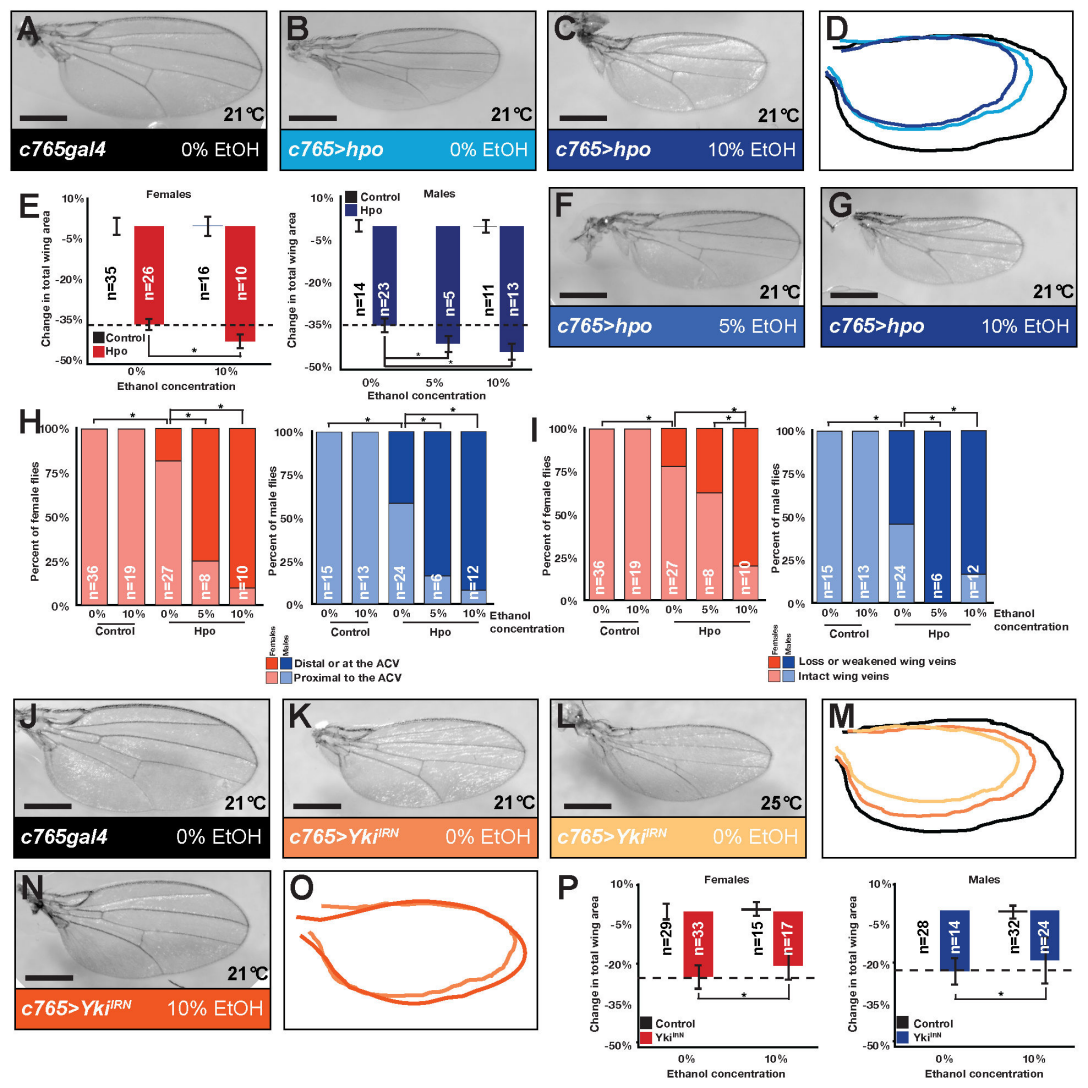
Ethanol promotes Hippo signaling in *Drosophila*. (A) Control wing. Representative wings over-expressing *hpo* reared on (B) 0% ethanol and (C) 10 % ethanol. (D) Tracings of wings in A-C. (E) Quantification of the effects of ethanol treatment on wing area (normalized to control wing treated with 0% ethanol). Significant area effects were seen at 5% in some but not all experiments. * indicates p=0.00004738091994 (males, 5%), p=0.0000003789435 (females, 10%), and p=5.50153E-11 (males, 10%). (F-G) Wings from flies reared on (F) 5% and (G) 10% ethanol showed increased patterning abnormalities. (H) Quantification of the effects of ethanol on proximal-distal positioning of the intersection of L2 and L3. Comparing control and Hpo over-expressing wings at 0% ethanol, * indicates p=0.0132429375674621 (females) and p=0.00003467107 (males). Comparing Hpo over-expressing wings at 0% to 5%, * indicates p=0.0000391195433611(females), p=0.03843393 (males). Comparing Hpo over-expressing wings at 0% to 10%, * indicates p=0.0000000059140932 (females) and p=0.000442676 (males). (I) Quantification of weakening and loss of segments within L2, L3, and L4 longitudinal veins. Comparing control and Hpo over-expressing wings at 0% ethanol, * indicates p=2.45092E-22 (females) and p=0.00000659349 (males). Comparing Hpo over-expressing wings at 0% to 5%, * indicates p=0.02424 (males). Comparing Hpo over-expressing wings at 5% to 10%, * indicates p=0.00550172 (females). Comparing Hpo over-expressing wings at 0% to 10%, * indicates p=0.00001108737(females) and p=0.04258 (males). (J) Control wing. (K-L) Representative wings undergoing RNAi to *yki*. Obvious wing size reduction is observed at 21°C (K) which increases at the higher temperature of 25°C. (M) Overlay of tracings of wings in J-L. (N) Representative wing undergoing RNAi to *yki* reared on 10% ethanol. (O) Overlay of tracings of wings in (K) and (N). (P) Quantification of the effects of ethanol treatment on wing area at 21°C (normalized to control wing treated with 0% ethanol). * indicates p=0.00433037 (females), p=0.030839921 (males). Wings shown are from females.

*w/UAS* *dcr2; engal/+* (Figure 1A-C,G, Figure 2A-B)
*w/UAS* *dcr2; engal/+; UAS hpo* ^*IRT*^/+ (Figure 1D-E,G)
*y* *w* *eyFLP; FRT42D* *pW+ UbiGFP/FRT42D hpo* ^*MGH1*^ (Figure 1H-K)
*w/UAS* *dcr2; engal/+; UAS GFP/+* (Figure 1L-O)
w*; c765gal4/+* (Figure 1P, Figure 2C,E-F, Figure 3A,E,H-J, P)
*w; c765gal4/UAS Myc* (Figure 1Q-S)
*w/UAS dcr2; engal/+; UAS Mer* ^*IRT*^/+ (Figure 2A)
*w/UAS dcr2; engal/*+; *UAS ex* ^*IRT*^ */*+ (Figure 2B)
w*; c765gal4/UAS wts* ^*IRN*^ */*+ (Figure 2C)
w*; engal/+* (Figure 2D)
w*; engal4/*+; *UAS ykiV5/*+ (Figure 2D)
w*; c765gal4/UAS FLAGyki* (Figure 2E)
w*; c765gal4/UAS ykiS168AGFP.HA* (Figure 2F)
w*; c765gal4/UAS hpo* ^*WT*^ */*+ (Figure 3B-I)
w*; c765gal4/UAS yki* ^*IRN*^ (Figure 3K-P)

### Quantification

Mosaic eyes were visually scored as: “+” for mild over-representation of mutant tissue (white) compared to wild-type tissue (red), “++” for moderate over-representation, and “+++” for strongest over-representation. To rule out unintentional observer bias, trials were conducted blind with lab members scoring eyes without knowledge of genotypes. Adult wings were mounted, photographed, and traced to give pixel area. For *engal4* wings, we measured area posterior to vein L4. For *c765gal4*, total wing area is shown. Area comparisons utilized T-tests; mosaic eye and wing abnormality comparisons utilized chi-squared tests. The data shown in the figures are representative experiments which have been performed independently a minimum of three times.

## Results and Discussion

### Ethanol enhances tissue overgrowth associated with loss of the Hippo tumor suppressor

In an ongoing screen to elucidate the link between alcohol and cancer, we used the Gal4/UAS system [51] and the FLP/FRT system of mosaic analysis [49] to modulate levels of conserved growth regulators, tumor suppressors, and oncogenes in various tissues (eye, wing, etc.) in *Drosophila* exposed to a range of dietary ethanol. Wings from control flies (*en>dcr2*) reared on food containing 1-10% ethanol generally showed no size increase, although consistent with previous reports [21] there were small but statistically significant size decreases in some cases (Figure 1A-B). Early in the wing screen, we identified the *hpo* tumor suppressor as a strong hit (Figure 1C-G). RNAi to *hpo* in the posterior wing increased size of this posterior compartment by more than 30% (Figure 1C-D,G). Posterior wing overgrowth was statistically significantly further increased by about 10% in flies reared on food containing 2-5% ethanol (Figure 1D-G) compared to *hpo* RNAi alone. 

To establish if ethanol-mediated enhancement of tissue overgrowth was a wing-specific phenomenon or a more general tissue response, we examined the effects of dietary ethanol on *hpo* mutant eye tissue. Mosaic eyes containing control homozygous white wild-type tissue and homozygous red wild-type tissue show roughly equal white and red tissue. Mosaic eyes containing tissue homozygous for *hpo* ^MGH1^, a strong hypomorphic allele, show over-representation of mutant tissue (white) compared to wild-type tissue (red) [35] in a range of over-representation from mild (“+” = more white than red, about 65% of the eyes, Figure 1H) to moderate (“++” = strongly more white than red, about 20% of eyes, Figure 1I) to severe (“+++” = almost all white, about 10-15% of the eyes, Figure 1J). Ethanol doses of 1-10% decreased the percentage of eyes in the mild “+” category and increased the percentage of eyes that were scored as moderate and severe (“++” or “+++”) (Figure 1K). At 5% ethanol, approximately 50% of eyes were severe (compared to less than 15% reared on 0% ethanol); at 7.5% ethanol, this increased to over 60% of eyes. 

We find it unlikely that RNAi off-target effects or alcohol regulation of the GAL4/UAS system cause this phenomenon because (1) ethanol did not affect Gal4/UAS-mediated expression of a *GFP* transgene (Figure 1L-O), and (2) alcohol enhanced over-representation of *hpo* ^MGH1^ mutant eye tissue (Figure 1H-K), a genetic system that used neither GAL4/UAS or RNAi tools. Moreover, these data indicate that this phenomenon is not specific to wing tissue but may represent a general response of *hpo*-deficient tissue to ethanol. 

The ability of alcohol to enhance overgrowth was not universal. Over-expressing the oncogene Myc in the wing (Figure 1Q) promotes wing overgrowth compared to a control wing (Figure 1P). Dietary ethanol did not enhance this overgrowth (Figure 1R-S). 

### Alcohol enhances organ overgrowth associated with expanded and warts but not Merlin or yorkie

To establish if enhancement of wing overgrowth applied to other components of the Hippo Pathway, we examined knockdown of upstream tumor suppressor components *Mer* and *ex*. Alcohol did not enhance overgrowth upon *Mer* RNAi in the posterior wing (Figure 2A), but statistically significantly enhanced overgrowth upon *ex* RNAi (Figure 2B). Our data could reflect interaction with alcohol specific for Ex-directed signaling through the pathway or could result from the complicated way in which upstream inputs direct pathway outputs. Signaling through the pathway is complex and not strictly linear; for example, Ex promotes signaling through Hpo [48] and also binds and inhibits Yki directly [52]. Loss of *Mer* or *ex* singly has distinct phenotypes from loss of core components *hpo* or *wts* [53]. However, simultaneous loss of *Mer* and *ex* phenocopies loss of *hpo* or *wts* in other contexts [48]. The interaction between alcohol and *ex* knockdown but not *Mer* knockdown can be further resolved as future work in the field elucidates how upstream factors activate the pathway to define distinct biological outputs.

To address downstream components, we examined knockdown of *wts* and over-expression of *yki*. RNAi to *wts* in the posterior wing with *engal4* led to such overgrowth that wing folding prevented accurate quantification. We therefore used weaker pan-wing driver *c765gal4*. Ethanol enhanced wing overgrowth from RNAi to *wts* in females (Figure 2C). 

Over-expressing *yki* in the posterior wing or across the whole wing promotes tissue overgrowth. We tested conditions that led to overgrowth similar to loss of *hpo* seen in Figure 1 for wild-type *yki* transgene *UAS-ykiV5*. Surprisingly, ethanol did not reproducibly enhance ykiV5-mediated overgrowth (Figure 2D). Therefore we tested a distinct wild-type *yki* transgene, *UAS-FLAGyki*. As with RNAi to *wts*, overgrowth produced by the *FLAGyki* transgene using *engal4* was too extensive to quantify, so we assessed overgrowth with *c765gal4*. Consistent with our findings for *ykiV5*, dietary ethanol did not enhance *FLAGyki-*mediated overgrowth (Figure 2E). Because wild-type versions of *yki* would be subject to inhibition by endogenous Hippo signaling, we also tested the transgene *UAS ykiS168AGFP.HA*. The S168A mutation cannot be phosphorylated by Wts at the 14-3-3 site, so is insensitive to Wts-induced inactivation via translocation out of the nucleus [54]. Dietary ethanol did not enhance the overgrowth due to *ykiS168AGFP.HA* expression (Figure 2F)*.*

If alcohol-mediated enhancement of overgrowth upon loss of *ex*, *hpo*, or *wts* occurs via interaction with Yki protein or its targets, we would predict alcohol to enhance Yki over-expression-induced overgrowth. Therefore, our genetic interaction studies suggest that alcohol may interact with Hippo signaling at or downstream of *hpo* and *wts*, possibly parallel to *yki*. This is particularly unexpected because signaling through Yki is reported to be crucial to overgrowth from loss of Hippo Pathway tumor suppression. Our findings may reveal a role for Yki-independent Hippo Pathway targets in promoting growth upon alcohol exposure. Alternatively, alcohol may be acting in a less straightforward way to regulate Yki protein not revealed by our wild-type and mutant Yki over-expression studies.

### Alcohol enhances Hpo over-expression phenotypes but not Yki loss phenotypes

How does alcohol enhance overgrowth due to loss of *ex, hpo*, and *wts*? A trivial explanation would be that alcohol further impairs signaling through the pathway. This explanation would predict that alcohol should therefore suppress the phenotypes of over-activating the pathway, such as by over-expressing *hpo* itself. In contrast, we observed that dietary ethanol enhanced *hpo* over-expression phenotypes. Over-expressing *hpo* in the wing reduced wing size and disrupted wing patterning (Figure 3B, quantified in 3E, 3H-I) compared to controls (Figure 3A). Flies reared in 5% ethanol-containing food showed a trend of enhanced wing size reduction while flies reared in 10% ethanol-containing food showed significantly enhanced wing size reduction (Figure 3C-E). Because knockdown experiments in Figure 1 do not eliminate all Hippo signaling, the increased Hippo pathway activation induced in the presence of 10% ethanol may explain the perplexing result that 10% ethanol doses did not enhance wing overgrowth or as strongly enhance eye mutant tissue over-representation upon Hippo loss as lower doses (Figure 1E-G, K). Exposure to 5% and 10% ethanol significantly enhanced mis-patterning caused by Hippo over-expression (Figure 3F-I). 

How can we reconcile that alcohol (1) enhanced overgrowth from impaired Hippo Pathway tumor suppression but (2) also enhanced the phenotype of *hpo* over-expression? Although in seeming conflict with our earlier results, the ability of ethanol to enhance *hpo* over-expression phenotypes is consistent with previous reports in the literature using *in vitro* systems. Hpo kinases Mst1 and Mst2 were identified in mammalian systems as stress response kinases [55] and are activated in cultured cells by oxidative stress [56-58]. Alcohol promotes oxidative stress; our findings may represent a validation of those *in vitro* effects in a physiological setting. 

Does this enhancement of Hippo-induced wing size reduction occur through further downregulation of *yki*? If so, we would expect alcohol to also enhance the wing size reduction of knocking down (but not knocking out) *yki* because presumably the increased Hippo signaling would act to further down-regulate the Yki protein produced. Similar to *hpo* over-expression, RNAi to *yki* across the entire wing results in a smaller wing (Figure 3K-M) compared to a control wing (Figure 3J). Parallel exposure of flies to doses of 10% ethanol (a dose at which we saw effects on *hpo* over-expression phenotypes of wing patterning and wing size) did not enhance the wing size reduction due to RNAi of *yki* (Figure 3N-P). This may suggest that that alcohol further enhances Hpo-mediated growth regulation through targets other than *yki.*

### A role for Hippo Pathway Tumor Suppression in response to stress?

**Figure 4:**
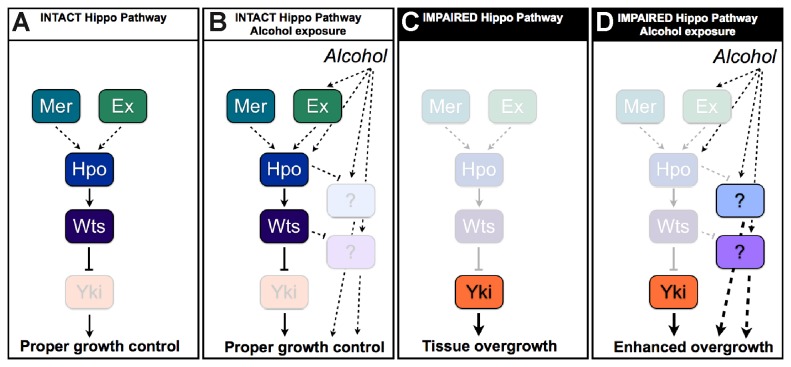
Model for alcohol interaction with the Hippo Pathway. As established, when Hippo signaling is intact (A), Yki is properly regulated and tissues maintain proper growth control. We propose that alcohol promotes activation of the pathway at or upstream of Hippo to target alcohol-relevant growth-promoting activities (B) to maintain proper growth control upon alcohol exposure. When Hippo signaling is impaired (C), Yki becomes overactive and promotes tissue overgrowth. We propose that when Hippo signaling is impaired in the presence of alcohol (D), Yki acts to promote overgrowth as established, and additional alcohol-relevant targets of the pathway act to promote further overgrowth.

If Hippo signaling is activated by alcohol as part of a stress response, it seems reasonable to speculate that Hippo signaling is acting to prevent some of the deleterious effects of alcohol. If this is the case, then impairing Hippo signaling in the presence of alcohol would mean those deleterious effects of alcohol would take place. Taking our findings into account with this logic, we propose that in addition to tissue homeostasis regulated by Hippo signaling under normal conditions (Figure 4A), alcohol does not promote the overgrowth of *Drosophila* eye and wing tissues in part because alcohol promotes activation of Hippo signaling (Figure 4B). However, in contexts where Hippo signaling is impaired and tissue undergoes established Yki-dependent overgrowth (Figure 4C), we propose alcohol can then also act to promote additional tissue overgrowth, possibly by interacting with Yki-independent targets of Hippo signaling (Figure 4D) because *yki* over-expression is not sufficient to recapitulate this phenomenon. 

The ability of alcohol to promote Hippo signaling in flies and for oxidative stress to promote Hippo signaling in mammalian systems [55-58] suggests there may be a highly conserved role for stress to activate the Hippo Pathway. This raises interesting questions regarding the origins of Hippo signaling to respond to alcohol. We speculate that activation of Hippo signaling by alcohol developed in a common ancestor as a mechanism to maintain proper patterning and growth regulation even upon exposure to environmental stressors; maintenance of this mechanism may have been selected for in species like *Drosophila* that develop in the presence of alcohol, such as on fermenting fruit or other species that encounter environmental exposure to related stressors, to prevent stress-mediated tissue overgrowth.

Our findings that alcohol enhances both Hippo Pathway loss of function and over-expression phenotypes are consistent with a role for dietary alcohol (i.e. whole animal exposure) to have specific effects on target organs undergoing Hippo Pathway modulation. This could be particularly relevant in prescribing lifestyle changes and for designing therapies for patients depending on whether or not their tumors maintain intact Hippo signaling. Moreover, our findings suggest that impaired Hippo Pathway tumor suppression may underlie the pathogenesis of specific alcohol-mediated cancers. Notably, there is striking overlap between Hippo-associated cancers [27-34] and alcohol-associated cancers, including colorectal, liver, and breast cancers [59-64]. 

Yki is the best-characterized target of the Hippo Pathway; its homologs YAP and TAZ are widely accepted to play an important role in cancer. Therefore, we were surprised that alcohol did not enhance tissue overgrowth upon *yki* over-expression. Alcohol may act in a novel way to promote Yki accumulation or activity. Indeed, alcohol promoted Hippo signaling which normally antagonizes *yki*; this could have masked alcohol-mediated enhancement of wild-type *yki* phenotypes and resulted in the lack of observed interaction between dietary ethanol and wild-type *yki* over-expression (Figure 2D). However (1), we did not observe any significant phenotypic enhancement of over-expressing *hpo* at dietary alcohol concentrations of 2% (not shown), where we did not see interactions between *yki* over-expression and ethanol and (2) alcohol did not enhance the organ size reduction caused by knockdown of *yki*. Therefore, we believe the simplest model to explain our data is that in the range of alcohol concentrations tested in our study, alcohol interacts with Yki-independent targets of Hpo and/or Wts. Our report may represent another context of Hippo Pathway functions that are Yki-independent including reported roles in F-actin regulation [65] and polarity [66]. Moreover, controversial reports propose YAP acts as a breast cancer tumor suppressor [67-69]. Thus, Hippo Pathway targets parallel to YAP could be particularly relevant to alcohol-mediated breast cancers. 
